# LncRNA HAGLR silencing inhibits IL-1β-induced chondrocytes inflammatory injury via miR-130a-3p/JAK1 axis

**DOI:** 10.1186/s13018-023-03661-4

**Published:** 2023-03-15

**Authors:** Yunzhou Zuo, Changjun Xiong, Xuewen Gan, Wei Xie, Xiaokang Yan, Yanzhao Chen, Xugui Li

**Affiliations:** Department of Orthopedics, The Affiliated Hospital of Wuhan Sports University, No. 279 Luoyu Road, Hongshan District, Wuhan, 430079 China

**Keywords:** LncRNA HAGLR, Osteoarthritis, miR-130a-3p/JAK1 axis

## Abstract

**Background:**

Osteoarthritis (OA), the most common form of arthritis, is accompanied by destruction of articular cartilage, development of osteophyte and sclerosis of subchondral bone. This study aims to explore whether lncRNA HAGLR can play a role in OA, and further clarify the potential mechanism.

**Material and methods:**

StarBase and luciferase reporter assay were applied for predicting and confirming the interaction between lncRNA HAGLR, miR-130a-3p and JAK1. The levels of lncRNA HAGLR and miR-130a-3p were analyzed using quantitative reverse transcription PCR (qRT-PCR). The proliferation, cytotoxicity and apoptosis of CHON-001 cells were evaluated by MTT, lactate dehydrogenase assay (LDH) and Flow cytometry (FCM) analysis, respectively. Moreover, expression of cleaved Caspase3 protein were determined by Western blot assay. The release of inflammatory factors (TNF-α, IL-8, and IL-6) was detected by ELISA.

**Results:**

lncRNA HAGLR directly targets miR-130a-3p. Level of lncRNA HAGLR was substantially higher and miR-130a-3p level was memorably lower in IL-1β stimulated CHON-001 cells than that in Control group. Furthermore, lncRNA HAGLR silencing alleviated IL-1β induce chondrocyte inflammatory injury, as evidenced by increased cell viability, reduced LDH release, decreased apoptotic cells, inhibited cleaved-Caspase3 expression, and reduced secretion of secretion of inflammatory factors. However, miR-130a-3p-inhibitor reversed these findings. We also found miR-130a-3p directly targeted JAK1 and negatively regulated JAK1 expression in CHON-001 cells. In addition, JAK1-plasmid reversed the effects of miR-130a-3p mimic on IL-1β-induced chondrocytes inflammatory injury.

**Conclusion:**

Silencing of lncRNA HAGLR alleviated IL-1β-stimulated CHON-001 cells injury through miR-130a-3p/JAK1 axis, revealing lncRNA HAGLR may be a valuable therapeutic target for OA therapy.

## Introduction

OA, the most common arthritis, is characterized by joint pain, deformation and limited movement. With the growth of population age, the incidence rate of OA increases year by year. According to statistics, about 3 million newly diagnosed OA cases occur every year, which is closely related to the significant incidence rate, mortality and increased medical burden of the middle-aged and elderly [1]. The literature shows that OA is related to many factors, including aging, traumatic inflammation, metabolic disorder and genetic factors [2, 3]. At present, the main treatment of OA is to reduce the weight bearing and excessive range of motion of joints, thus delaying the progress of the disease [4]. The current commonly used anti-inflammatory and analgesic drugs can reduce or control symptoms, but it cannot be taken for a long time [5]. Therefore, timely diagnosis and reasonable treatment of OA are expected to improve its poor prognosis.

Non coding RNAs have been reported to play key roles in musculoskeletal conditions [6–8]. LncRNAs are a non-coding RNA with a length of more than 200 nucleotides [9]. The abnormal expression or function of lncRNA is closely related to the occurrence of human diseases, including breast cancer [10], liver diseases [11] and many other major diseases. Studies have shown that lncRNA plays a certain role in regulating the pathological changes of OA. Luo et al. found that knockdown of lncRNA MFI2-AS1 inhibits LPS-induced OA progression through miR-130a-3p/TCF4 [12]. Zhang et al. found that LncRNA MALAT1 promotes osteoarthritis by modulating miR-150-5p/AKT3 axis [13]. LncRNA HAGLR was reported to be vital regulator in multiple diseases, including myocardial I/R injury [14], dermatomyositis [15] and femoral neck fracture healing [16]. However, the function of lncRNA HAGLR in OA remains unclear, and the specific mechanism of lncRNA HAGLR in OA needs to be deeply illustrated.

MiRNA is a kind of small RNA with a length of about 20–24 nucleotides, which has many important regulatory functions in cells [17]. In recent years, the role of miRNA in the development of diseases has attracted extensive attention [18]. Studies have shown that some miRNA expression changes may have therapeutic potential for OA. For instance, Cai et al. revealed that miR-27a promotes the autophagy and apoptosis of IL-1β stimulated-articular chondrocytes in OA through PI3K/AKT/mTOR signaling [19]. Besides, report from Cao et al. demonstrated that decreased miR-214-3p activates NF-kB pathway and aggravates OA progression [20]. MiR-130a-3p, a preclinical therapeutic target, was evidenced to be significant regulators in Crohn's disease and neuropathic pain [21, 22], but the exact mechanism of miR-130a-3p in OA remains unclear.

In recent years, IL-1β stimulated CHON-001 cells are widely used in the study of OA in vitro [23–25]. In this study, IL-1β simulated CHON-001 cells are used as an in vitro model for the study of OA. Therefore, our study aims to explain the potential functions of lncRNA HAGLR and miR-130a-3p in IL-1β simulated CHON-001 cells and elucidate their potential mechanisms. Our results suggest that lncRNA HAGLR may play a role in osteoarthritis by regulating the expression of miR-130a-3p. We identified lncRNA HAGLR as a new therapeutic target for patients with OA, which provides a theoretical basis for the treatment of OA.

## Materials and methods

### Cells culture

CHON-001 cells were acquired from the ATCC and grown in DMEM (Thermo Fisher), supplemented with 10% FBS and 100 U/ml penicillin/streptomycin in humidified 5% CO_2_ at 37˚C. CHON-001 cells were treated with different concentrations of IL-1β for 12 h to establish injury model.

### Dual-luciferase reporter assay

StarBase was applied to investigate the relationship between miR-130a-3p and lncRNA HAGLR or JAK1. As miR-130a-3p and lncRNA HAGLR for example, the lncRNA HAGLR luciferase reporter gene plasmids were used to illustrate the latent targets of lncRNA HAGLR and miR-130a-3p. MiR-130a-3p was proved to be a potential target of lncRNA HAGLR. For reporter activity assay, lncRNA HAGLR wild-type or mutant plasmids together with miR-130a-3p mimic or mimic control were co-transfected into 293 T cells using Lipofectamine 2000 (Invitrogen) following the protocol for 24 h. Dual-Luciferase Reporter Assay System was applied to assess the luciferase activity.

### qRT-PCR analysis

The isolation of RNA from CHON-001 cells was obtained with the TRIZol reagent (Thermo Fisher) following the guidance. Then the cDNA was synthesized from the collected RNA using the PrimeScript RT reagent kit (Thermo Fisher) following the guidance. cDNAs were assessed using qPCR with the SYBR Premix Ex TaqTM II kit to examine the LncRNA HAGLR, miR-130a-3p and GAPDH. The results were calculated using 2^−ΔΔCt^ method.

### Cell transfection

The CHON-001 cells were induced by control-siRNA, HAGLR-siRNA, inhibitor control, miR-130a-3p-inhibitor, mimic control, miR-130a-3p mimic, control-plasmid, or JAK1-plasmid using Lipofectamine® 3000 reagent (Thermo Fisher) for 24 h referring to the protocol. The cells were treated with IL-1β (10 ng/ml) for 12 h and then subjected to subsequent experiments.

### MTT assay

After transfected for 24 h or treated with IL-1β for 12 h, CHON-001 cells were cultured into 96-well plates at 37 °C. Then, cells were dealt with 10 μl MTT solution and cultivated for 4 h. Then 100 μL DMSO was applied to dissolve lysate for 10 min. Finally, cell viability was assessed by measuring the absorbance at 570 nm by a multifunctional plate reader according to the protocol.

### LDH assay

LDH released from cells were detected by LDH-Cytotoxicity Assay Kit (Sigma). Briefly, the CHON-001 cells were transfected for 24 h or treated with IL-1β for 12 h. Then the supernatant of CHON-001 cells were collected. Then the culture supernatant and cell lysates were cultivated with LDH reaction mixture following the protocols for 15 min. The absorbance was detected at 490 nm and LDH release was calculated with a microplate reader (BioTek, USA).

### FCM assay

After digested with trypsin without EDTA and washed with PBS, the CHON-001 cells were assessed using the Annexin-V/propidium iodide (PI) Apoptosis Detection Kit (Tianjin Sungene Biotech Co., Ltd). The cells were gently mixed and were cultivated for 20 min at room temperature without light. Finally, apoptotic cells were determined using Flow cytometer (BD Technologies) and analyzed with FlowJo.

### Western blot assay

The proteins from CHON-001 cells were extracted using RIPA buffer (Beyotime) and valued using a bicinchoninic acid assay kit (Beyotime). Then averaged proteins were separated by 10% SDS-PAGE, and transferred to PVDF membrane. Then the membrane was blocked in 5% skim milk for 2 h at room temperature and incubated in specific antibodies against β-actin or cleaved-Caspase3 (1:1000 dilution) overnight at 4 °C. Later on, the membrane was incubated in secondary antibodies for 1 h. Finally, signals were developed by ECL detection system reagents (Pierce Biotechnology) according to the synopsis.

### ELISA assay

After transfected for 24 h or treated with IL-1β for 12 h, the supernatant were collected from CHON-001 cells and the secretion levels of IL-6, IL-8 and TNF-α in supernatant were detected by ELISA kits (BD biosciences) following the protocol. Then, the optical density (OD) value at 450 nm was read on Multiscan Spectrum (MD, USA).

### Statistical analysis

All the above experiments were performed three times. All results were presented as the mean ± SD and analyzed by using GraphPad Prism 6.0. Differences among groups were estimated using one-way analysis of variance (ANOVA) and student’s t-test. *P < 0.05, and **P < 0.01 indicated statistically as significant difference.

## Results

### LncRNA HAGLR directly targets miR-130a-3p

**Fig. 1:**
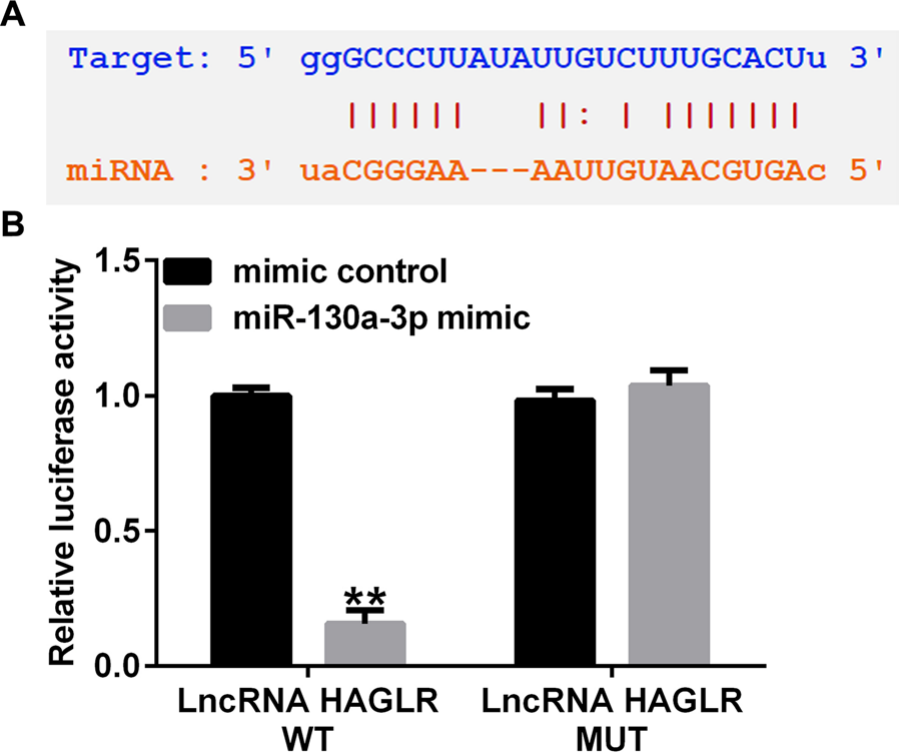
LncRNA HAGLR directly sponges miR-130a-3p. **A** Predicted binding site of miR-130a-3p in lncRNA HAGLR. **B** Association between lncRNA HAGLR and miR-130a-3p was analyzed by Dual-luciferase reporter assay. **P < 0.01 versus Mimic control

We firstly analyzed whether lncRNA HAGLR targets miRNAs as endogenous contend RNAs, results from StarBase suggested a latent lncRNA HAGLR binding site on miR-130a-3p (Fig. 1A). Further findings confirmed that over-expression of miR-130a-3p reduced the luciferase activity in lncRNA HAGLR-WT transfected cells, while there was no significant effects on lncRNA HAGLR-MUT transfected cells (Fig. 1B). Our data indicated that miR-130a-3p directly interacted with lncRNA HAGLR.

### LncRNA HAGLR was over-expressed and miR-130a-3p was down-regulated in IL-1β-stimulated CHON-001 cells

**Fig. 2:**
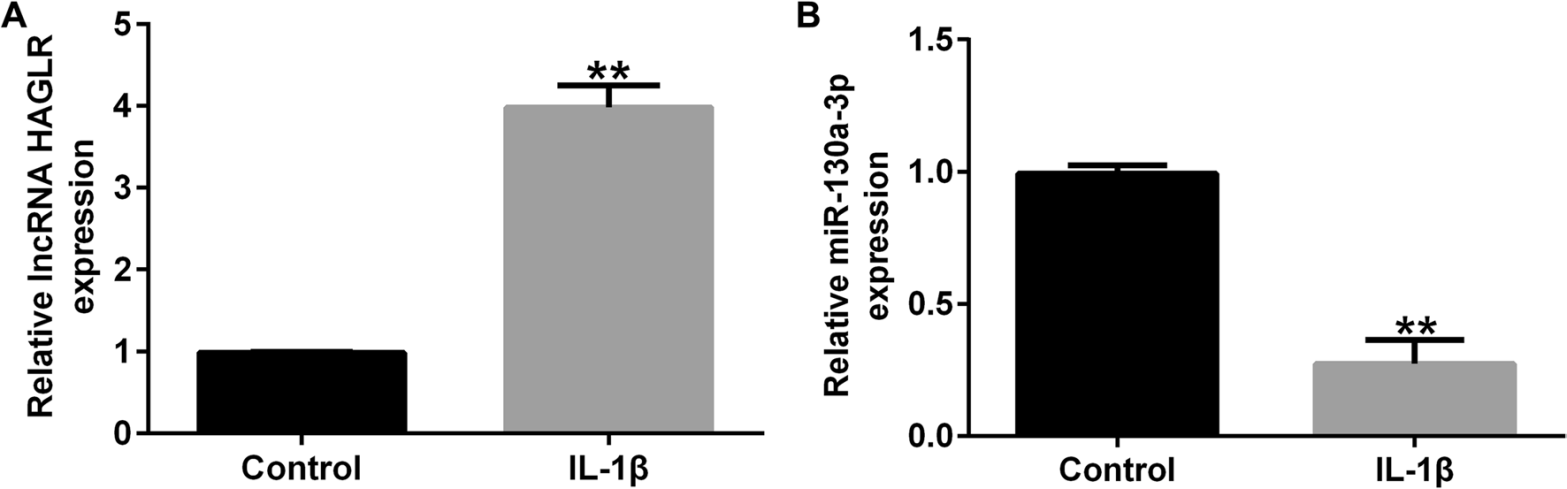
Expression of lncRNA HAGLR and miR-130a-3p in IL-1β induced CHON-001 cells. qRT-PCR analysis of lncRNA HAGLR (**A**) and miR-130a-3p (**B**) in IL-1β induced CHON-001 cells. **P < 0.01 versus control

CHON-001 cells were exposed to 10 ng/ml IL-1β to induced inflammatory damage of chondrocytes for 12 h. After that, levels of lncRNA HAGLR and miR-130a-3p in CHON-001 cells was determined. As presented in Fig. 2A and B, the level of lncRNA HAGLR was substantially higher in IL-1β induced chondrocyte than that in control group. In addition, we observed miR-130a-3p was down-regulated in IL-1β stimulated CHON-001 cells. Our findings suggested that lncRNA HAGLR and miR-130a-3p might function in osteoarthritis.

### LncRNA HAGLR negatively regulated miR-130a-3p level in CHON-001 cells

**Fig. 3:**
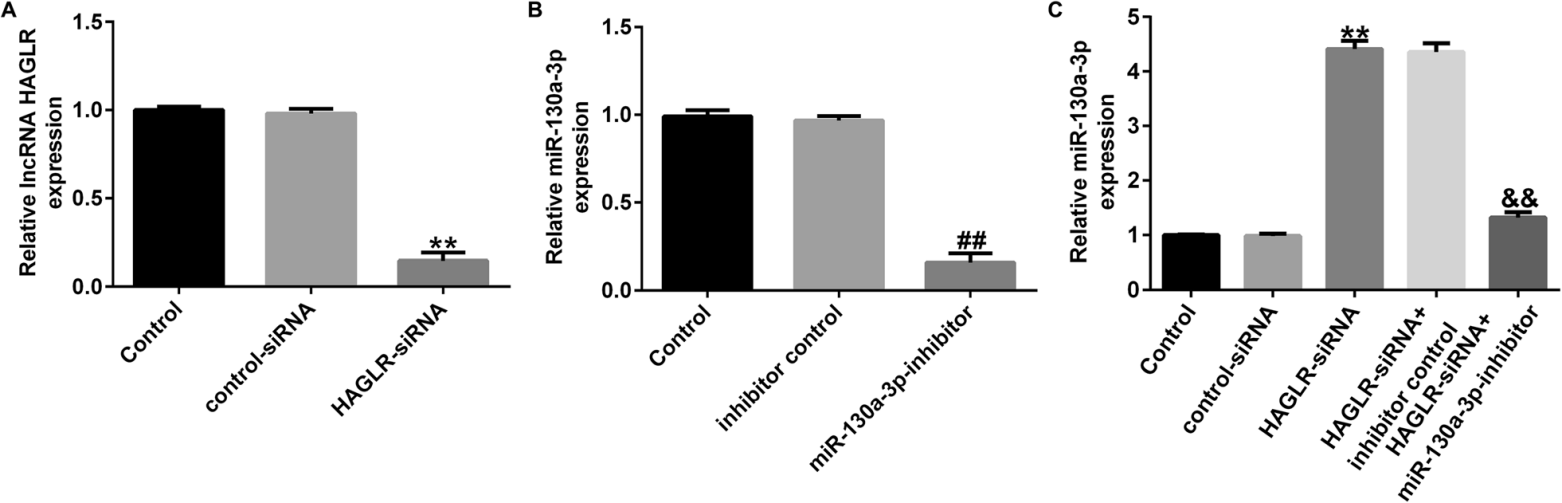
MiR-130a-3p inhibitor reversed the effects of HAGLR-siRNA on miR-130a-3p expression. **A** Levels of lncRNA HAGLR were evaluated using qRT-PCR analysis. **B** Expression of miR-130a-3p in miR-130a-3p-inhibitor or inhibitor control transfected CHON-001 cells using qRT-PCR. **C** qRT-PCR analysis of miR-130a-3p mRNA levels in HAGLR-siRNA or miR-130a-3p-inhibitor transfected CHON-001 cells. **P < 0.01 versus Control-siRNA; ##*P* < 0.01 versus Inhibitor control; &&*P* < 0.01 versus HAGLR-siRNA + inhibitor control

We also revealed the correlation between lncRNA HAGLR and miR-130a-3p in osteoarthritis, control-siRNA, HAGLR-siRNA, inhibitor control, or miR-130a-3p-inhibitor were transfected into CHON-001 cells. Results from Fig. 3A and B revealed that HAGLR-siRNA substantially reduced HAGLR expression, and the level of miR-130a-3p was lower in miR-130a-3p-inhibitor transfected cells than that in inhibitor control group. Furthermore, our observations showed that HAGLR-siRNA signally enhanced miR-130a-3p expression in CHON-001 cells, while this promotion was eliminated by miR-130a-3p-inhibitor (Fig. 3C). According to these observations, we concluded that lncRNA HAGLR negatively regulated miR-130a-3p level in osteoarthritis.

### Silence of lncRNA HAGLR alleviated IL-1β-stimulated chondrocytes inflammatory injury by regulating miR-130a-3p

**Fig. 4:**
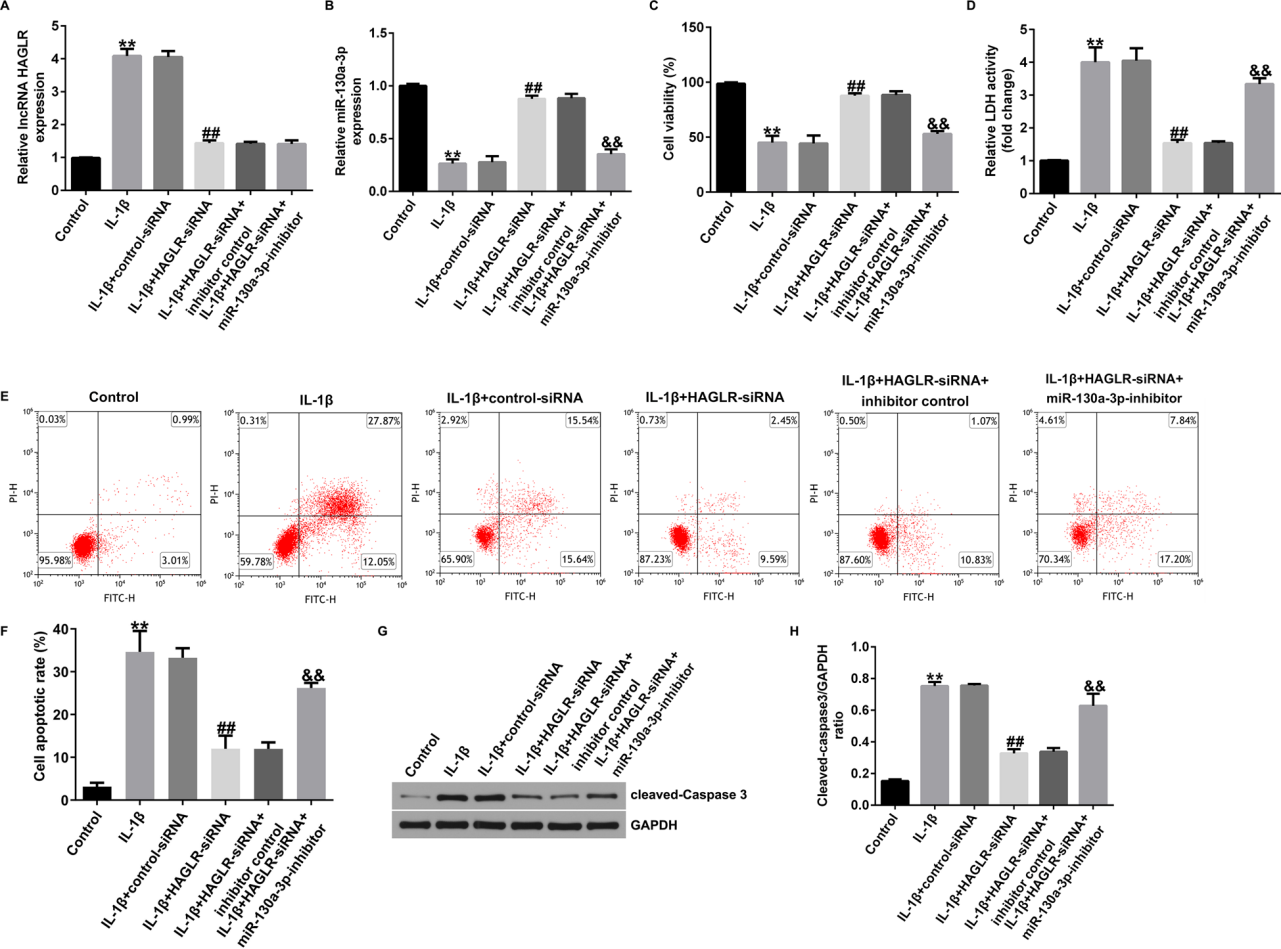
Effects of lncRNA HAGLR-siRNA on IL-1β induced CHON-001 cells viability, LDH release and apoptosis. Expression of lncRNA HAGLR (**A**) and miR-130a-3p (**B**) were determined using qRT-PCR. **C** MTT assay of cell viability. **D** Analysis of LDH release. **E** Cell apoptosis were assessed by flow cytometry analysis. **F** Quantification of apoptotic cells. **G** Protein expression of cleaved-Caspase3. **H** Quantization of Caspase3 expression. **P < 0.01 versus Control; ##*P* < 0.01 versus IL-1β + control-siRNA; &&*P* < 0.01 versus IL-1β + HAGLR-siRNA + inhibitor control

Having illustrating the relevance between lncRNA HAGLR and lncRNA HAGLR, we further explained the biological functions of CHON-001 cells after control-siRNA, HAGLR-siRNA, miR-130a-3p-inhibitor or inhibitor control treatment. Data suggested that HAGLR-siRNA reversed the effects of IL-1β on lncRNA HAGLR and miR-130a-3p expression, as confirmed by suppressed lncRNA HAGLR level, as well as increased miR-130a-3p expression. However, there was no obvious change on lncRNA HAGLR expression, and miR-130a-3p was down-regulated in miR-130a-3p-inhibitor transfected CHON-001 cells (Fig. 4A and B).

We also found that IL-1β led to suppressed cell viability (Fig. 4C), accelerated LDH release (Fig. 4D), increased apoptotic cells (Fig. 4E and F), and enhanced cleaved-Caspase3 expression (Fig. 4G and H), while we observed the opposite findings in HAGLR-siRNA transfected cells. However, these observations were successfully reversed by miR-130a-3p inhibitor, indicating that down-regulation of miR-130a-3p could relieve IL-1β-stimulated CHON-001 cells viability and apoptosis through regulating miR-130a-3p.

### Silence of lncRNA HAGLR relieved IL-1β-induced inflammatory response by regulating miR-130a-3p

**Fig. 5:**
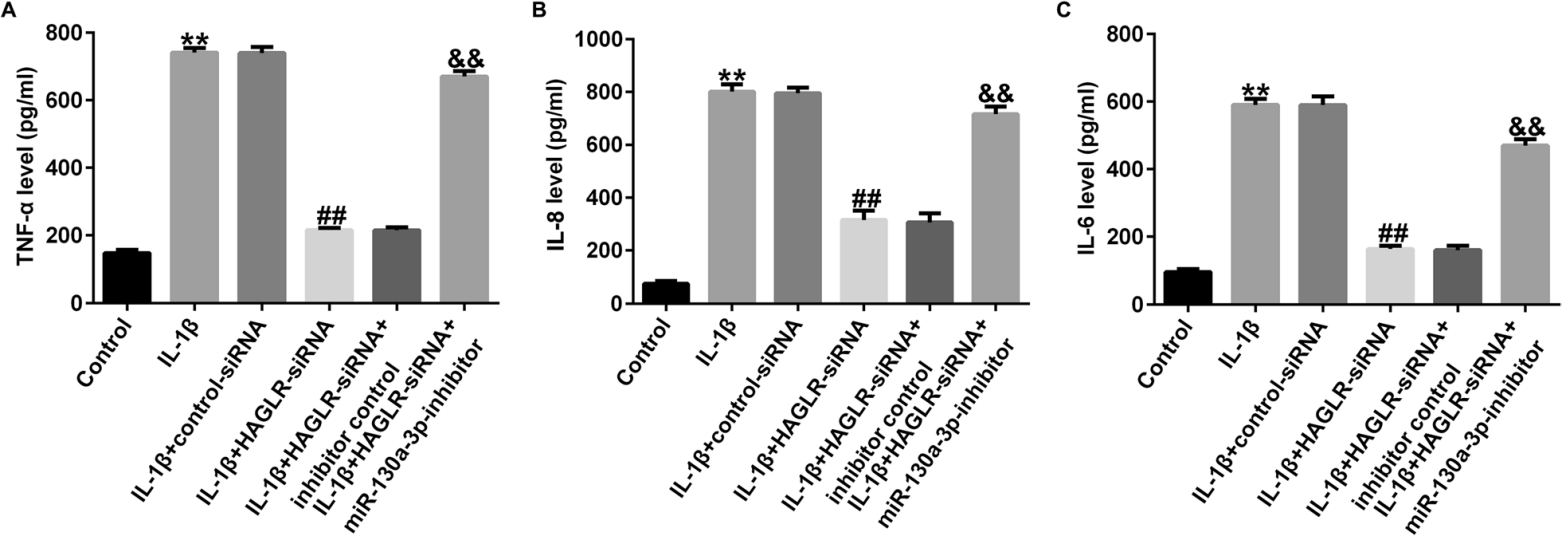
Effects of lncRNA HAGLR-siRNA on inflammatory cytokine secretion in IL-1β induced CHON-001 cells. Levels of TNF-α (**A**), IL-8 (**B**), IL-6 (**C**) and were determined using ELISA assay. **P < 0.01 versus Control; ##*P* < 0.01 versus IL-1β + control-siRNA; &&*P* < 0.01 versus IL-1β + HAGLR-siRNA + inhibitor control

Moreover, we illustrated the roles of lncRNA HAGLR and miR-130a-3p in inflammatory factors release in chondrocytes, including TNF-α, IL-8, and IL-6. ELISA assay demonstrated that the levels of inflammatory elements were dramatically improved in IL-1β-treated cells, and silence of HAGLR notably suppressed inflammatory response, compared to control-siRNA group (Fig. 5A–C). However, we found the opposite results in miR-130a-3p-inhibitor transfected cells, revealing that silence of lncRNA HAGLR alleviated IL-1β-treated inflammatory response through targeting miR-130a-3p.

### JAK1 directly interacted with miR-130a-3p

**Fig. 6:**
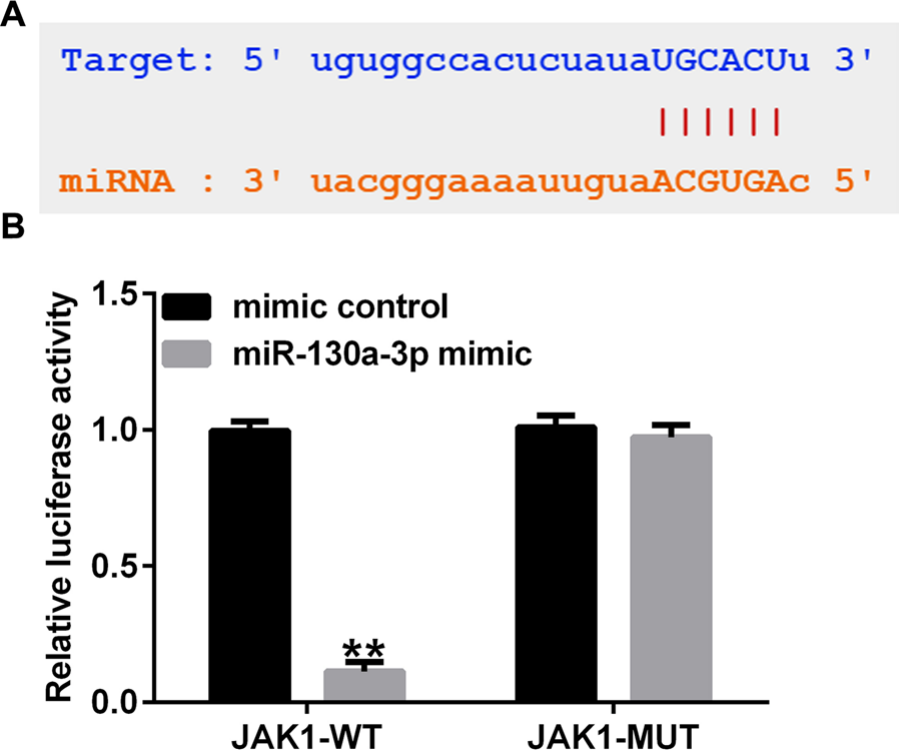
JAK1 is a direct target of miR-130a-3p. **A** A schematic of JAK1 binding site in miR-130a-3p 3’-UTR. **B** Luciferase activities were analyzed using Dual luciferase reporter assay. **P < 0.01 versus Mimic control

To further explore the underlying mechanisms of miR-130a-3p in CHON-001 cells, TargetScan assay was applied for predicting the latent target gene of miR-130a-3p. As displayed in Fig. 6A and B, JAK1 was a candidate target of miR-130a-3p (Fig. 6A). Dual luciferase reporter gene system further showed that miR-130a-3p mimic memorably suppressed the luciferase activity of JAK1 3’-UTR wild-type, while the luciferase activity of JAK1 3’-UTR mutant type had no obvious change, revealing that JAK1 directly interacted with miR-130a-3p (Fig. 6B).

### MiR-130a-3p negatively regulates JAK1 expression in CHON-001 cells

**Fig. 7:**
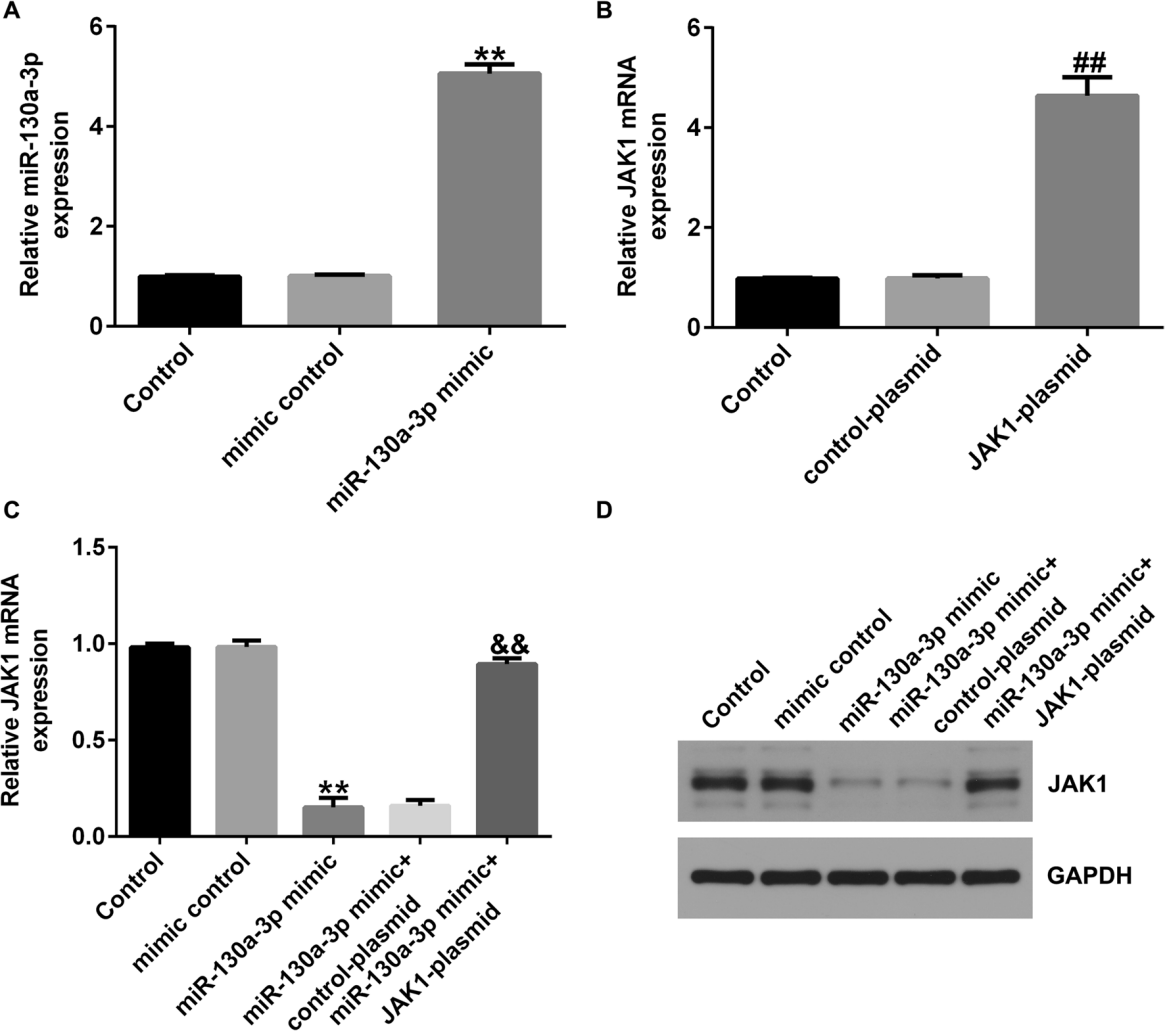
MiR-130a-3p negatively regulates JAK1 in CHON-001 cells. **A** qRT-PCR analysis of miR-130a-3p mRNA levels in miR-130a-3p mimic or mimic control transfected cells. Evaluation of JAK1 level in JAK1-plasmid **B** transfected cells using qRT-PCR assay. **C** and **D** The mRNA and protein level of JAK1 in miR-130a-3p mimic + JAK1-plasmid transfected cells. **P < 0.01 versus Mimic control; ##*P* < 0.01 versus Control-plasmid; &&*P* < 0.01 versus miR-130a-3p mimic + control-plasmid

Then we explained the regulatory effect of miR-130a-3p on JAK1 expression in CHON-001 cells, mimic control, miR-130a-3p mimic, control-plasmid, or JAK1-plasmid were transfected into cells for 24 h. Findings from Fig. 7A presented that miR-130a-3p was up-regulated in miR-130a-3p mimic transfected cells, compared to control-plasmid group. Moreover, JAK1 level was significantly improved in JAK1-plasmid transfected CHON-001 cells (Fig. 7B), and down-regulated in miR-130a-3p mimic group, as opposed to mimic control group. Nevertheless this suppression was eliminated after JAK1-plasmid treatment (Fig. 7C and D). In summary, we observed that miR-130a-3p negatively regulates JAK1 expression in CHON-001 cells.

### JAK1-plasmid reversed the effects of miR-130a-3p mimic on IL-1β-induced chondrocytes inflammatory injury

**Fig. 8:**
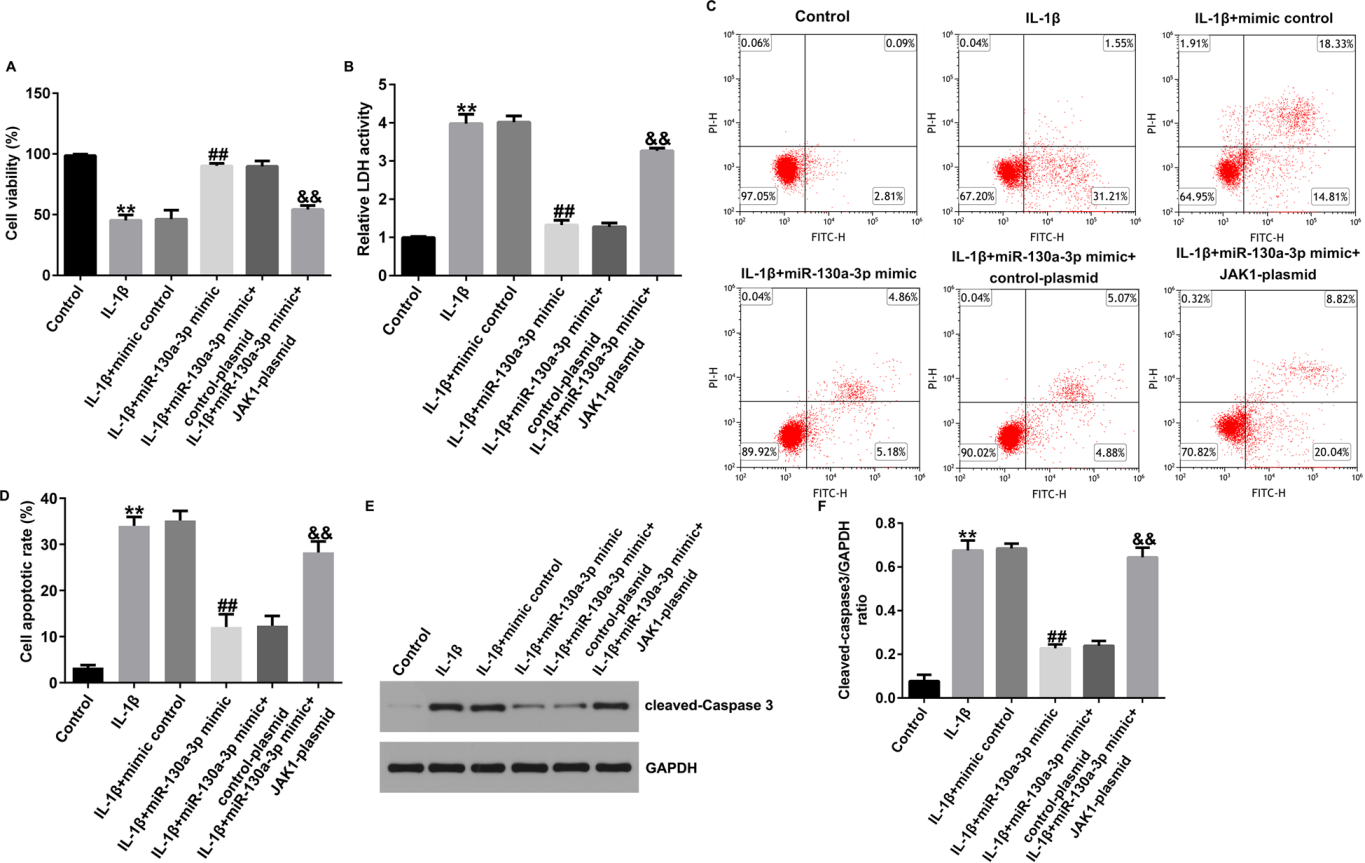
Effects of miR-130a-3p on IL-1β induced CHON-001 cells viability, LDH release and apoptosis. **A** MTT assay of cell viability. **B** Analysis of LDH release. **C** Apoptotic cells were evaluated by flow cytometry analysis. **D** Quantification of apoptotic cells. **E** Determination of cleaved-Caspase3 protein expression by western blot assay. **F** Quantization of Caspase3 expression. **P < 0.01 versus Control; ##*P* < 0.01 versus IL-1β + mimic control; &&*P* < 0.01 versus IL-1β + miR-130a-3p mimic + control-plasmid

Further biological functions of JAK1 and miR-130a-3p in osteoarthritis were illustrated. CHON-001 cells were exposed to mimic control, miR-130a-3p mimic, control-plasmid or JAK1-plasmid for 24 h. As displayed in Fig. 8, miR-130a-3p mimic reversed the roles of IL-1β in CHON-001 cells viability, LDH release and apoptosis cell, as confirmed by promoted cell viability (Fig. 8A), inhibited LDH release (Fig. 8B), decreased apoptotic cells (Fig. 8C and D), and suppressed cleaved-Caspase3 expression (Fig. 8E and F), while these findings were successfully reversed after JAK1-plasmid treatment. Our findings suggested that lncRNA HAGLR silencing inhibits IL-1β-induced chondrocytes inflammatory injury via miR-130a-3p/JAK1 axis.

### JAK1-plasmid reversed the effects of miR-130a-3p mimic on IL-1β-induced inflammatory release

**Fig. 9:**
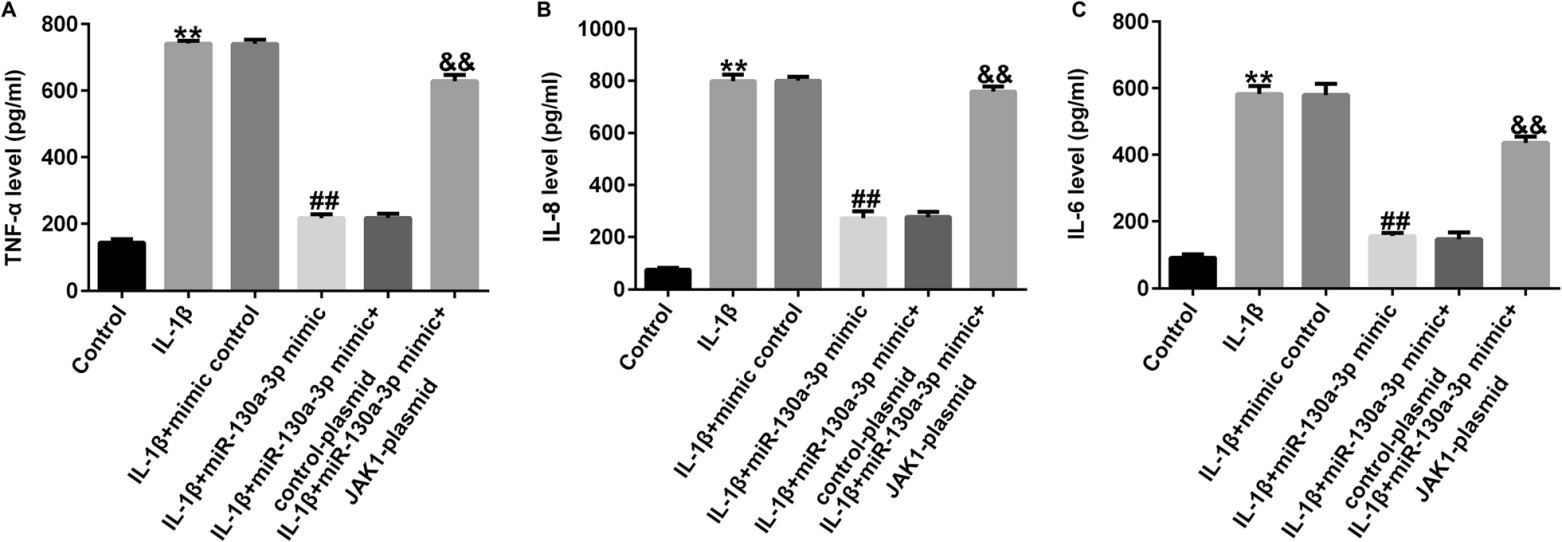
Effects of JAK1-plasmid on inflammatory cytokine secretion in IL-1β induced CHON-001 cells. Levels of TNF-α (**A**), IL-8 (**B**), IL-6 (**C**) were determined using ELISA assay. **P < 0.01 versus Control; ##*P* < 0.01 versus IL-1β + mimic control; &&*P* < 0.01 versus IL-1β + miR-130a-3p mimic + control-plasmid

Similarly, we explained the roles of JAK1 and miR-130a-3p in inflammatory release in CHON-001 cells. As presented in Fig. 9A–C, the secretion of TNF-α, IL-8, and IL-6 were prominently inhibited in miR-130a-3p mimic-stimulated CHON-001 cells, while this inhibition was eliminated by JAK1-plasmid.

## Discussion

OA, characterized by articular cartilage degradation, is a common joint disease worldwide, which is considered to be a worldwide public health problem. Increasing reports indicate that OA is caused by many factors, including strain, deformity and obesity [26]. Chondrocytes are the only cells found in articular cartilage and have been shown to play an important role in maintaining tissue homeostasis [27]. Recent studies have shown that lncRNA plays a potential role in OA treatment. For example, lncRNA THUMPD3-AS1 promoted chondrocytes proliferation and inflammatory response in OA [28]. In recent years, lncRNA HAGLR has been found to play a promoting role in various diseases [14–17]. However, the specific functions of lncRNA HAGLR in OA still unclear. Our research aims to focus on whether lncRNA HAGLR plays an important role in the development of OA by targeting miRNA.

Firstly, we found the target gene of lncRNA HAGLR and proved the relationship between miR-130a-3p and lncRNA HAGLR. Existing evidence indicates that IL-1β is increased in arthritic joints, which can cause chondrocyte apoptosis and inflammation [29, 30]. In this investigation, CHON-001 cells were exposed to 10 ng/ml IL-1β for 12 h to generate the OA model in vitro*.* To further clarify the roles of lncRNA HAGLR in OA, we evaluated the levels of lncRNA HAGLR and miR-130a-3p in chondrocytes. We found that lncRNA HAGLR was up-regulated and miR-130a-3p was low-expressed in IL-1β induced CHON-001 cells, which were consistent with other report, suggesting that lncRNA HAGLR potentially contributes to the development of OA by targeting miR-130a-3p. Numerous investigations have suggested that abnormal expression of lncRNA HAGLR may be instrumental in diseases therapy [15, 31]. Then control-siRNA, HAGLR-siRNA, inhibitor control, or miR-130a-3p-inhibitor was transfected into CHON-001 cells for 24 h. Our data revealed that lncRNA HAGLR negatively regulated miR-130a-3p level in CHON-001 cells. Evidence has been shown that dysregulation of lncRNAs is linked to their biological functions by targeting miRNAs in OA. Li et al. suggested that lncRNA LEMD1-AS1 relieves chondrocyte inflammation by targeting miR-944/PGAP1 in OA [32]. We further illustrated the latent mechanisms of lncRNA HAGLR in CHON-001 cells. Our observations were consistent with previous data shown that IL-1β led to inhibited cell viability and stimulated LDH release [33]. He et al. also shown that a high rate of apoptosis in chondrocytes could result in matrix degradation [34]. Results from flow cytometry assay further demonstrated that IL-1β led to increased apoptotic cells and enhanced cleaved-Caspase3 activity, while we found the opposite results in HAGLR siRNA transfected cells. However, after miR-130a-3p inhibitor transfection, all these data were successfully reversed, indicating that HAGLR silencing can alleviate IL-1β-stimulated CHON-001 cells viability and apoptosis by targeting miR-130a-3p.

Recent reports have pointed out the mechanism of miRNA in OA pathology, suggesting that miRNA affects the function of chondrocytes through the role of genes related to OA pathology (including proinflammatory cytokines, chemokines or some growth factors) [35]. Zheng et al. proved Butein inhibits IL-1β-induced inflammatory response in human OA chondrocytes [36]. Consistent with these results, our study shows that down-regulation of lncRNA HAGLR alleviated IL-1β-induced inflammatory response in CHON-001 cells, as confirmed by reduced IL-6, IL-8 and TNF-α level. However, we found the opposite results in miR-130a-3p-inhibitor transfected cells. Thus, blocking chondrocyte apoptosis and inflammatory response may be beneficial for OA treatment. Increasing evidences have verified that dysregulation of miRNAs is linked to their biological functions by targeting mRNAs in OA. Cao et al. suggested that decreased miR-214-3p activates NF-kappaB pathway and aggravates osteoarthritis progression [37]. We further explored the potential mechanisms of miR-130a-3p in CHON-001 cells. Based on bioinformatics tools, miR-130a-3p directly sponges JAK1 and its expression was negatively regulated by JAK1. JAK1/STAT3 and FOXM1 are key mediators of inflammatory response. Research shows the interaction between JAK1/STAT3 and FOXM1 may play a key role in the study of OA pathogenicity [38]. Besides, Xu et al. suggested that circUBXN7 suppresses cell proliferation and facilitates cell apoptosis in LPS-induced cell injury by sponging miR-622 and regulating the IL6ST/JAK1/STAT3 axis [39]. Similar results were observed in this study, we found that JAK1-plasmid partially reversed the influence on cells viability, LDH release, apoptosis, and inflammatory response, which caused by miR-130a-3p mimic.

According to the above investigation, HAGLR silencing inhibits IL-1β-induced cell apoptosis and inflammatory response in chondrocytes through miR-130a-3p/JAK1 axis, thus preventing OA progression. Therefore, lncRNA HAGLR may be considered as a promising biomarker for the treatment of OA. Additional in vivo explorations are needed to affirm the precise mechanism of lncRNA HAGLR in the development of OA.

## Data Availability

The datasets used and/or analyzed during the current study are available from the corresponding author on reasonable request.
