# Correction: LncRNA HAGLR silencing inhibits IL‑1β‑induced chondrocytes inflammatory injury via miR‑130a‑3p/JAK1 axis

**DOI:** 10.1186/s13018-025-05732-0

**Published:** 2025-04-15

**Authors:** Yunzhou Zuo, Changjun Xiong, Xuewen Gan, Wei Xie, Xiaokang Yan, Yanzhao Chen, Xugui Li

**Affiliations:** Department of Orthopedics, The Affiliated Hospital of Wuhan Sports University, No. 279 Luoyu Road, Hongshan District, Wuhan, 430079 China

**Correction : Journal of Orthopaedic Surgery and Research (2023) 18:203** 10.1186/s13018-023-03661-4.

In this article, Figs. 4 and 8 appeared incorrectly and have now been corrected in the original publication. For completeness and transparency, the incorrect and correct versions of Figs. 4 and 8 are displayed below.

Incorrect Fig. 4
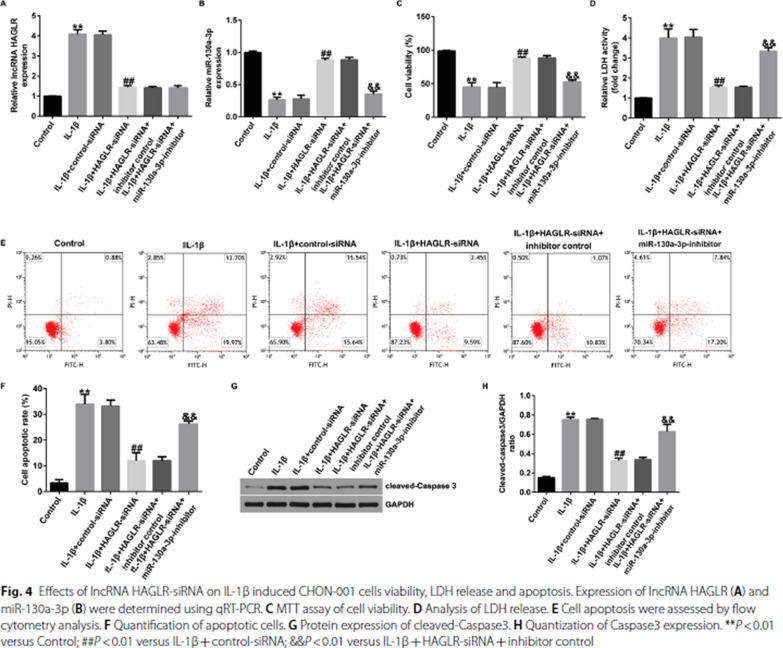


Correct Fig. 4

**Fig. 4:**
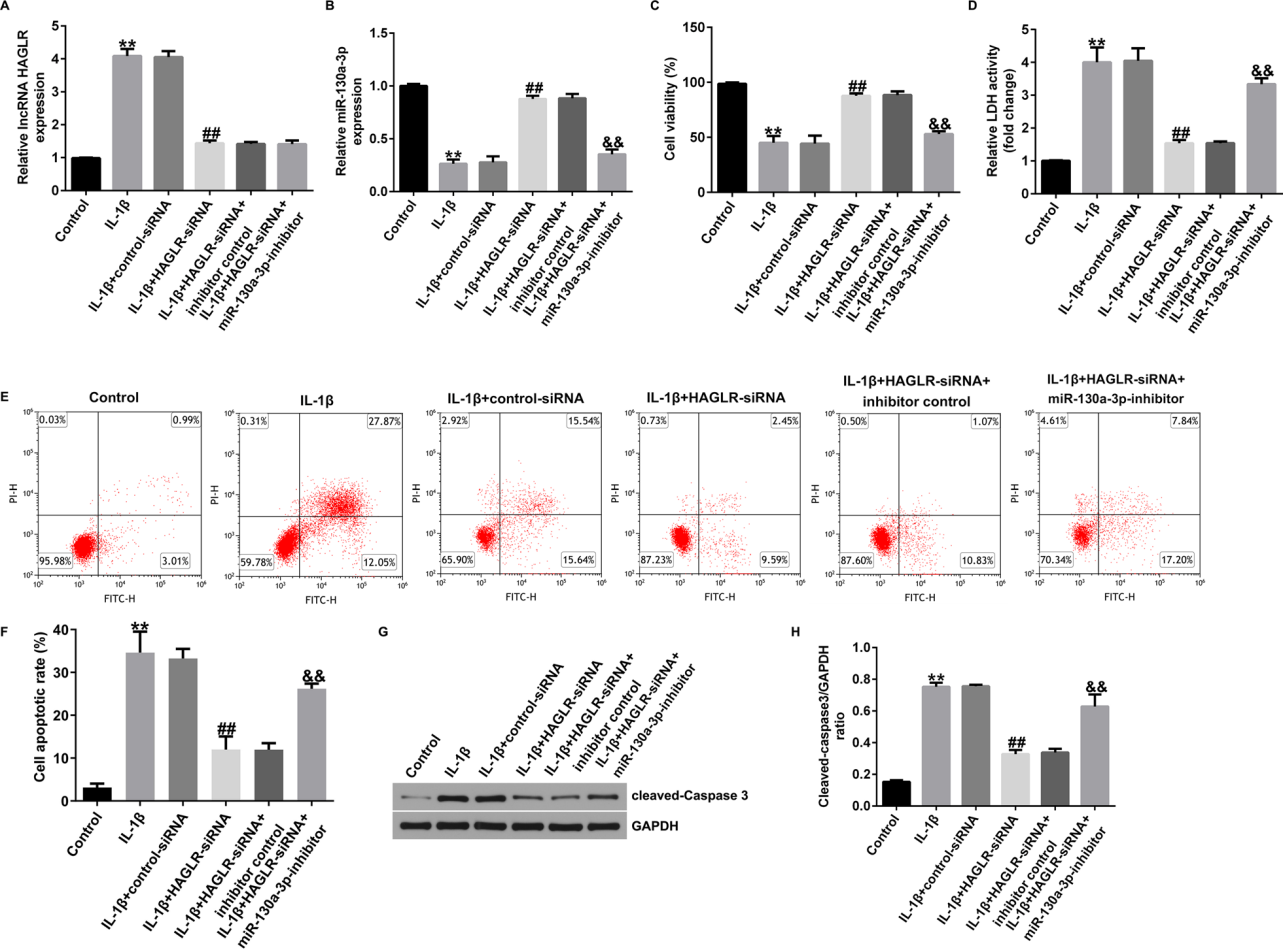
Effects of lncRNA HAGLR-siRNA on IL-1β induced CHON-001 cells viability, LDH release and apoptosis. Expression of lncRNA HAGLR (**A**) and miR-130a-3p (**B**) were determined using qRT-PCR. **C** MTT assay of cell viability. **D** Analysis of LDH release. **E** Cell apoptosis were assessed by flow cytometry analysis. **F** Quantification of apoptotic cells. **G** Protein expression of cleaved-Caspase3. **H** Quantization of Caspase3 expression. **P < 0.01 versus Control; ##*P* < 0.01 versus IL-1β + control-siRNA; &&*P* < 0.01 versus IL-1β + HAGLR-siRNA + inhibitor control

Incorrect Fig. 8
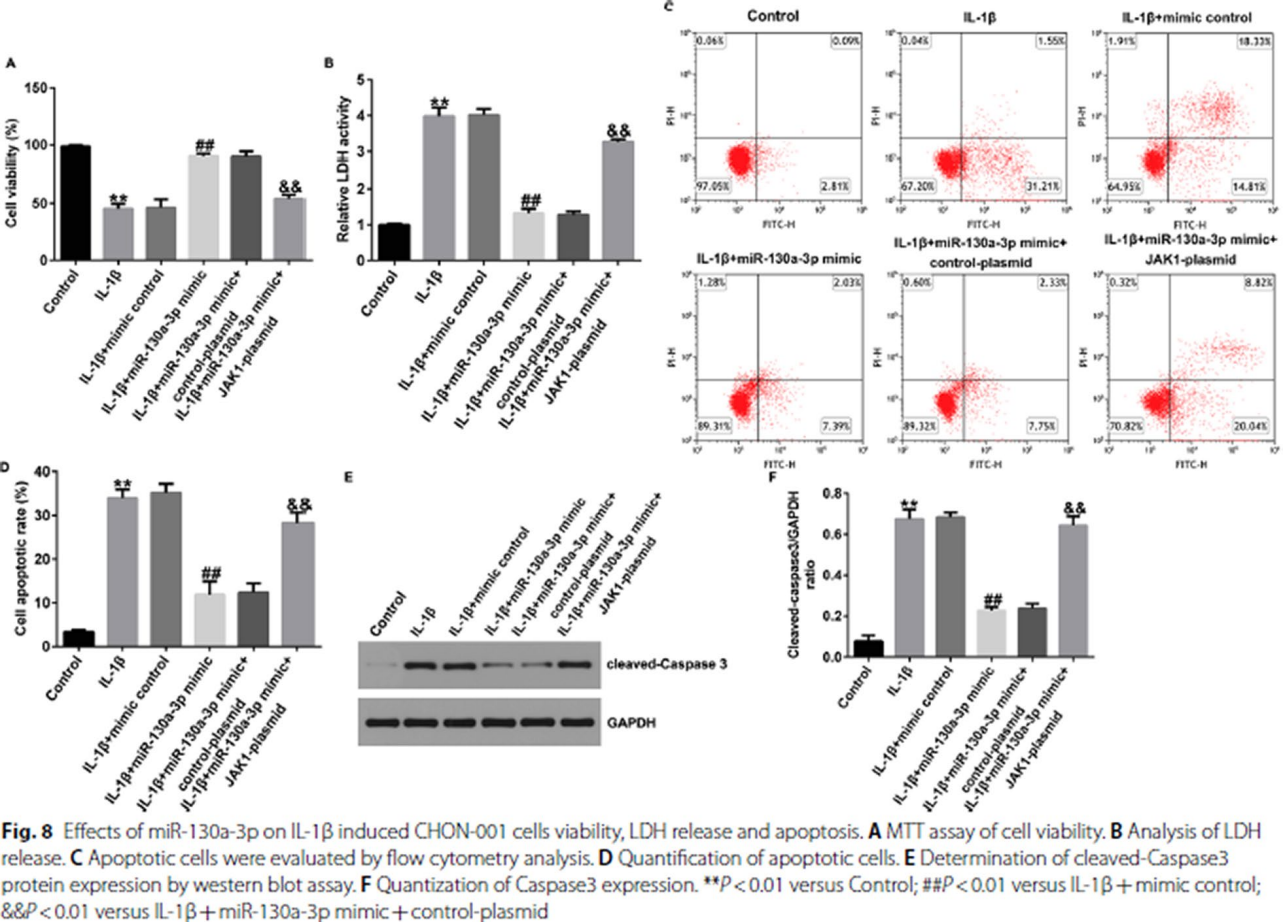


Correct Fig. 8

**Fig. 8:**
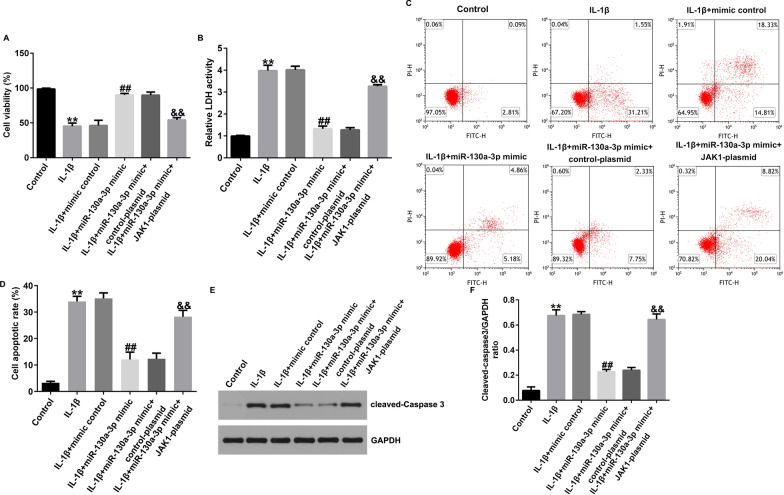
Effects of miR-130a-3p on IL-1β induced CHON-001 cells viability, LDH release and apoptosis. **A** MTT assay of cell viability. **B** Analysis of LDH release. **C** Apoptotic cells were evaluated by flow cytometry analysis. **D** Quantification of apoptotic cells. **E** Determination of cleaved-Caspase3 protein expression by western blot assay. **F** Quantization of Caspase3 expression. **P < 0.01 versus Control; ##*P* < 0.01 versus IL-1β + mimic control; &&*P* < 0.01 versus IL-1β + miR-130a-3p mimic + control-plasmid

The original article has been corrected.

